# Acute Psychosis and Dissociative Syndrome Following Binge Recreational Nitrous Oxide Use: A Case Report

**DOI:** 10.7759/cureus.116414

**Published:** 2026-09-17

**Authors:** Francisco Agostinho, Adriana Moutinho, Catarina K Câmara, Maria Beatriz Resende

**Affiliations:** 1 Psychiatry, Hospital Beatriz Ângelo, Loures, PRT

**Keywords:** acute psychosis, aripiprazole, case report, functional cobalamin deficiency, hyperhomocysteinaemia, neutropenia, nitrous oxide, recreational drug use, risperidone

## Abstract

Recreational nitrous oxide (N2O) inhalation is increasingly prevalent among young adults, but acute psychiatric presentations following short-term binge exposure remain poorly characterized. We report a case of a female in her early 20s with no prior history of psychotic illness who developed an acute psychotic and dissociative syndrome over three days at an outdoor music festival. Her presentation followed heavy recreational N2O and 3,4-methylenedioxymethamphetamine (MDMA) exposure combined with sleep deprivation. On admission, she exhibited profound disorientation, inability to recognize close family members, polymorphic delusions, passivity phenomena, and auditory and olfactory hallucinations, requiring involuntary psychiatric hospitalization. Diagnostic workup revealed normal brain and cervical spine magnetic resonance imaging (MRI) and a normal electroencephalogram (EEG) without epileptiform activity or slowing. Serum homocysteine was moderately elevated (25.2 µmol/L) alongside a low-normal vitamin B12 concentration (270 pg/mL), consistent with N2O-induced functional cobalamin deficiency; both markers normalized following parenteral vitamin B12 and oral folic acid supplementation. During inpatient stay, treatment with risperidone was followed by progressive neutropenia, which fully resolved after cross-titration to aripiprazole. She gradually regained full insight, was discharged on day 12, and remained free of psychotic symptoms at her three-week outpatient review. This case suggests that short-term binge N2O exposure, together with MDMA and sleep deprivation, may contribute to severe neuropsychiatric presentations without chronic use; it illustrates the value of measuring homocysteine rather than relying solely on serum B12 levels; and highlights potential hematological adverse effects during antipsychotic initiation.

## Introduction

Recreational nitrous oxide (N2O) use has increased steeply among young adults across Europe, driven by low cost, widespread commercial availability, and a common perception of harmlessness, particularly in festival and nightlife settings [1,2]. The neurotoxicity of N2O is classically attributed to functional cobalamin (vitamin B12) deficiency secondary to irreversible oxidation of the cobalt core of cobalamin, inactivating methionine synthase [3-5]. Clinically, this usually presents as subacute combined degeneration of the spinal cord or peripheral neuropathy following prolonged, heavy exposure over months [3-5].

Conversely, acute psychiatric presentations associated with N2O are far less characterized in the literature. A recent systematic review identified only 20 reported cases of psychosis attributed to N2O misuse, the vast majority of which occurred in men with chronic, escalating daily consumption over extended periods [6]. As N2O is rapidly eliminated through the lungs and is undetectable on routine urine toxicology screens, acute psychiatric manifestations are easily misattributed to primary psychotic disorders or co-ingested illicit stimulants, potentially missing a treatable toxic-metabolic contribution [6].

Here, we describe a case of a young female who developed a severe psychotic and dissociative syndrome after a single weekend of heavy N2O exposure, combined with 3,4-methylenedioxymethamphetamine (MDMA) use and severe sleep deprivation, in whom the metabolic signature of cobalamin inactivation was documented and reversed, and whose clinical course was further complicated by neutropenia during risperidone treatment.

## Case presentation

Patient information and history

A female in her early 20s presented to the emergency department with acute behavioral change. She lived independently, worked full-time, and shared custody of her young child. She had no significant medical history and took no regular medications. Her psychiatric history was notable only for brief psychological follow-up in early adolescence for oppositional behavior, mild, self-limiting postpartum depressive symptoms, for which prescribed antidepressants were never initiated, and mild anxiety, low mood, anhedonia, and insomnia over the preceding months attributed to relationship stress, which had subjectively resolved. A sibling had pubertal-onset epilepsy, but there was no family history of psychotic or bipolar disorders.

The patient reported episodic recreational alcohol, MDMA, and N2O use at social gatherings over two to three years, with increasing frequency and subjective tolerance. She reported past cannabis use that had ceased several years prior. Collateral accounts were discrepant: her mother denied any substance use, whereas a close friend reported weekly binge drinking, sporadic MDMA use, and frequent attendance at electronic music events over the preceding year.

Clinical presentation and emergency department course

The patient attended a four-day outdoor music festival with her friend starting on day -5. N2O inhalation on day -4 was initially suspected by her friend from a balloon found in her pocket, alongside reported MDMA use. Although precise individual exposure quantification remains difficult, the patient subsequently confirmed that her social group consumed six nitrous oxide canisters of unknown size over the festival days, with frequent and repeated inhalation throughout each day.

From day -3, her friend observed new-onset paranoid ideation (such as asking others to taste her water and refusing to use elevators) accompanied by marked restlessness despite minimal sleep (estimated at three hours across three consecutive nights). On day -1, during the journey home, she exhibited loud soliloquy and abnormal orofacial and ocular dyskinesias ("grimacing and strange movements of the eyes and mouth"). She visited a local emergency department but refused medical assessment. Following a brief period of sleep at home, she awoke unable to recognize her mother or state her own name, prompting emergency medical transport to a psychiatric emergency department on day 0 (Figure 1).

**Figure 1:**
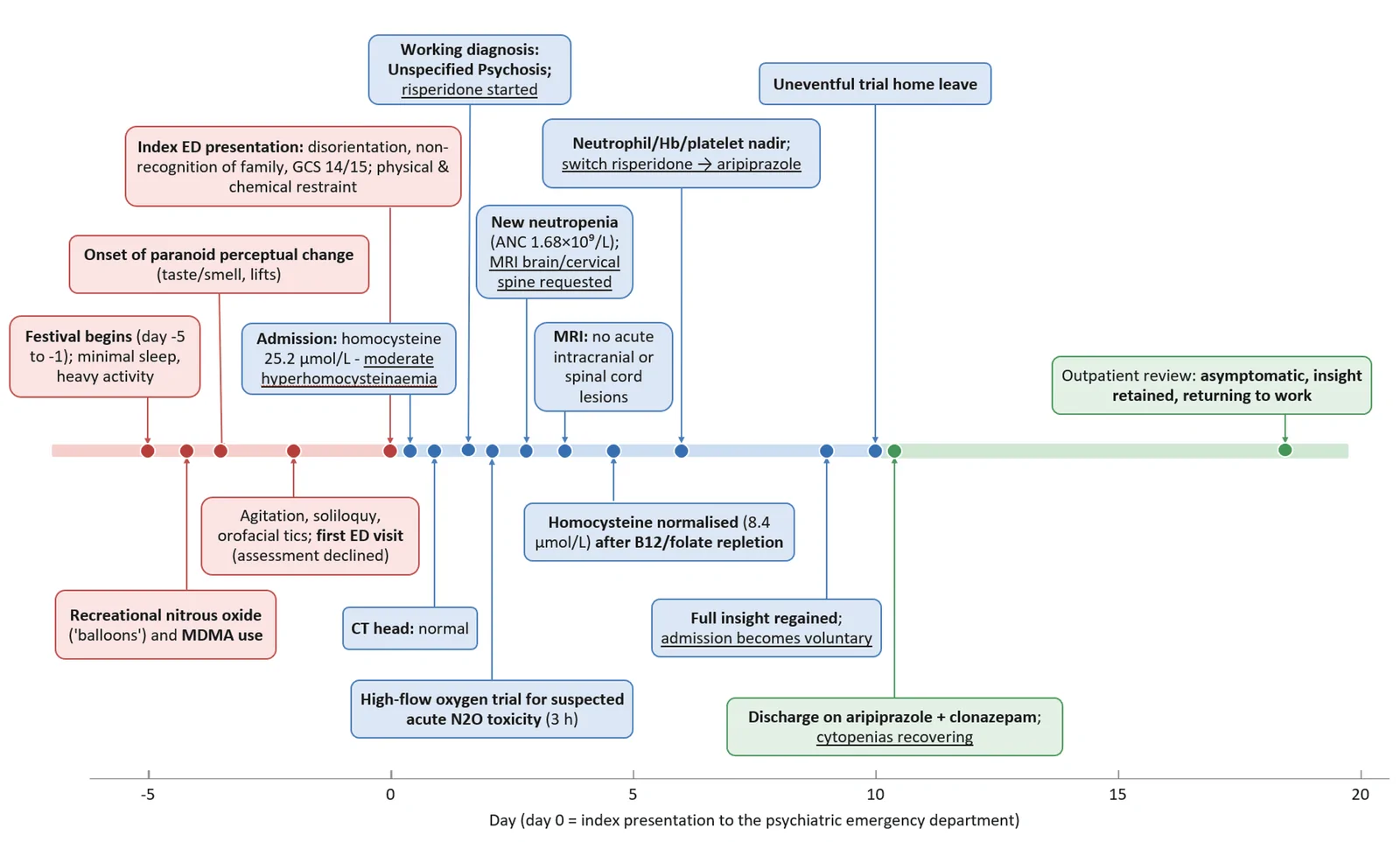
Timeline of clinical trajectory, diagnostic evaluations, and therapeutic interventions. Chronological overview of the patient's clinical course. The timeline details the onset of psychiatric symptoms, emergency department admission (day 0), baseline diagnostic evaluations, initiation of parenteral vitamin B12 repletion and antipsychotic cross-titration to aripiprazole, resolution of neutropenia and psychotic symptoms, and sustained recovery at the three-week outpatient evaluation. ANC: absolute neutrophil count; GCS: Glasgow Coma Scale; Hb: hemoglobin; MDMA: 3,4-methylenedioxymethamphetamine; N2O: nitrous oxide. Diagram created and arranged using Microsoft PowerPoint (Microsoft Corporation, Redmond, WA).

At triage on day 0, her Glasgow Coma Scale (GCS) score was 14 (baseline 15), and she was oriented only to person. She was easily rousable, haughty, quarrelsome, with non-syntonic contact, irritable mood, and inappropriate, sarcastic laughter. Her speech was partly prompted, repetitive, and poorly coherent, frequently losing track of the conversation without formal abnormalities of rate, volume, or syntax. Escalating agitation, repeated uncooperative attempts to leave, and complete lack of decision-making capacity required brief physical restraint, parenteral sedation, and involuntary admission under national mental health legislation.

Her mental status subsequently fluctuated between somnolence and agitation. Memory impairment was prominent: she failed to recognize photographs of her own child, yet immediately identified an aunt in another photograph. From day 3, poorly systematized polymorphic delusions emerged, encompassing persecutory, mystical, and control themes, including thought insertion, thought broadcasting, a Capgras-type misidentification of a treating physician, derealization, simple auditory hallucinations, and delusional interpretation of real olfactory stimuli. Insight was completely absent.

Investigations

Physical examination revealed that she remained apyrexial, normoglycemic, and hemodynamically stable apart from transient mild hypotension. Initial blood tests on day 0 showed normal full blood count parameters: hemoglobin = 14.4 g/dL, mean corpuscular volume (MCV) = 88.6 fL, absolute neutrophil count (ANC) = 3.25 × 10⁹/L, and platelets = 317 × 10⁹/L. Coagulation profile, serum electrolytes, calcium, magnesium, phosphate, thyroid function, creatine kinase, and C-reactive protein were all within reference ranges. Serum creatinine was marginally elevated at 1.01 mg/dL (estimated glomerular filtration rate = 78 mL/min/1.73 m²). Initial elevations in total bilirubin and lactate dehydrogenase were attributed to specimen hemolysis documented by the laboratory; repeat testing on day 6 yielded normal results.

Serological testing for human immunodeficiency virus (HIV), hepatitis B, hepatitis C, syphilis, blood ethanol, and serum β-human chorionic gonadotrophin were negative. Urinalysis on day 1 showed isolated nitrites without leukocyturia; repeat urinalysis on day 6 was entirely normal. An extended urine drug screen obtained on day 1 was positive only for therapeutic benzodiazepines administered in the hospital. This negative result did not exclude reported exposures, as urine collection occurred four days post-exposure (urinary MDMA is detectable for one to two days, and N2O is undetectable on standard urine toxicology panels). Non-contrast computed tomography (CT) of the head showed only mild maxillary sinus mucosal thickening.

Given clinical suspicion of N2O toxicity, serum vitamin B12 was drawn and measured at a low-normal level of 270 pg/mL (reference range: 197-771 pg/mL), with normal serum folate. Serum homocysteine, drawn on day 1 shortly after the initial dose of parenteral B12, was elevated at 25.2 µmol/L (reference range: 5-15 µmol/L), confirming moderate hyperhomocysteinemia (Figure 2). Magnetic resonance imaging (MRI) of the brain and cervical spine performed on day 5 demonstrated no parenchymal abnormalities, no cord signal changes, and no inverted V sign (Figure 3). An electroencephalogram (EEG) performed on day 5, requested due to fluctuating consciousness and a family history of epilepsy, demonstrated normal cerebral background activity without focal or generalized slowing, and no epileptiform discharges (Figure 4).

**Figure 2:**
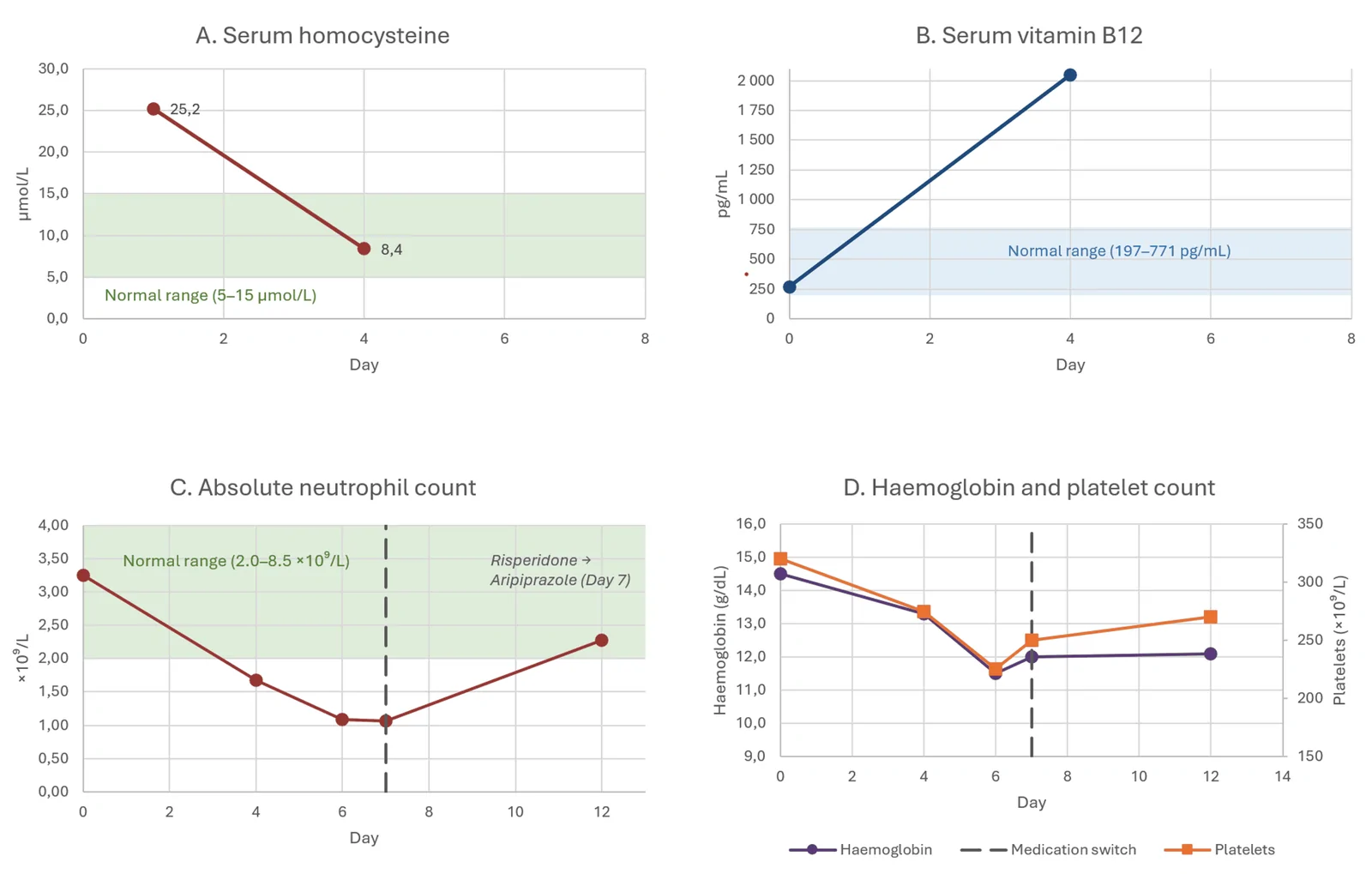
Serial biochemical and hematological parameters during hospitalization and follow-up. (A) Serum homocysteine concentrations shortly after the first dose and after three days of parenteral vitamin B12 and folic acid administration. (B) Serum vitamin B12 levels. (C) Absolute neutrophil count (ANC) trend showing the nadir on day 7 and recovery following cross-titration from risperidone to aripiprazole. (D) Hemoglobin and platelet count trajectories. Shaded areas represent laboratory reference ranges. Graph created and arranged using Microsoft Excel and Microsoft PowerPoint (Microsoft Corporation, Redmond, WA).

**Figure 3:**
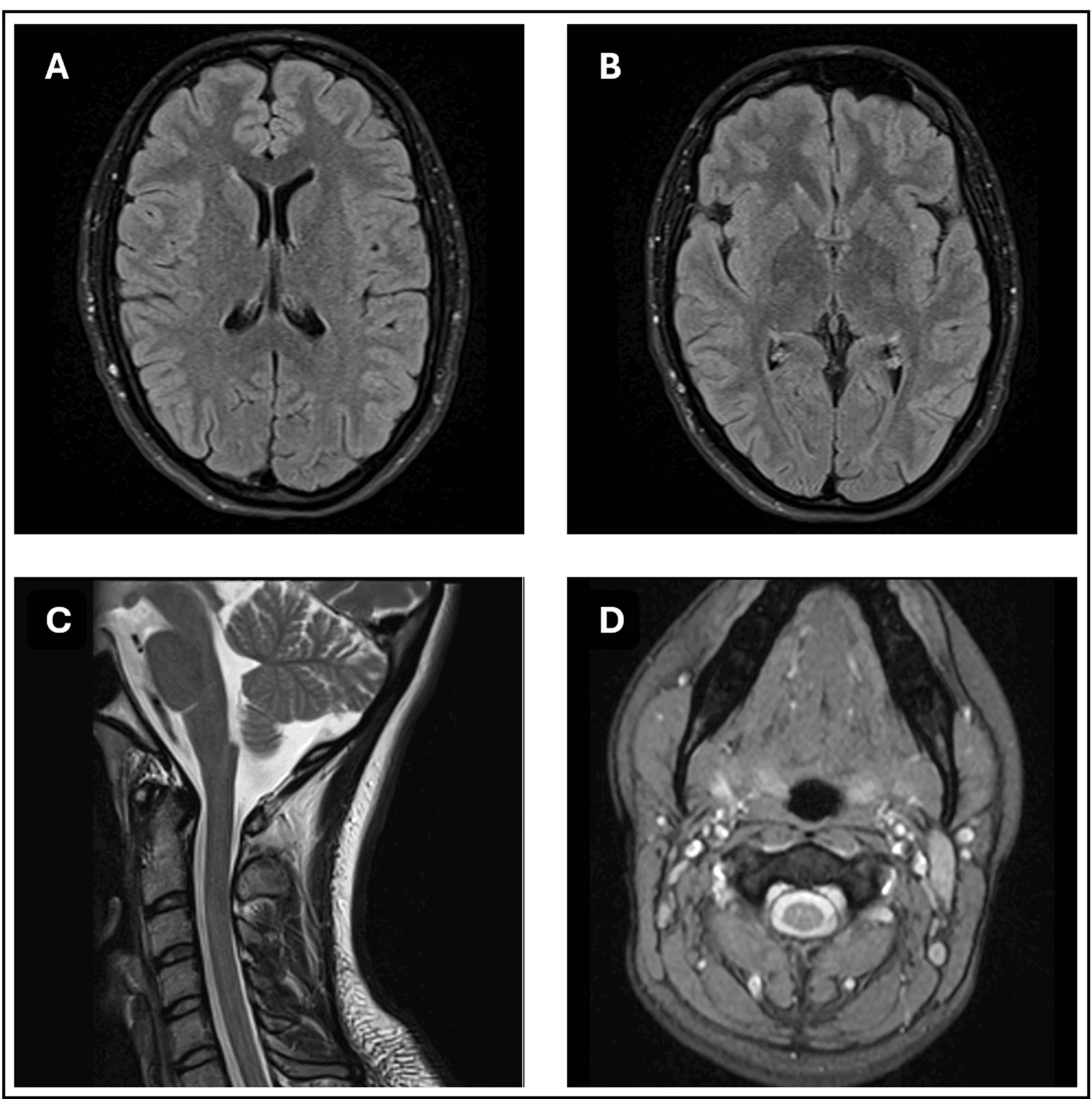
Brain and cervical spine magnetic resonance imaging (MRI). (A, B) Axial T2-weighted fluid-attenuated inversion recovery (FLAIR) brain MRI demonstrating normal cerebral parenchyma, intact basal ganglia, and preserved periventricular white matter, without evidence of toxic leukoencephalopathy or acute focal lesions. (C) Sagittal T2-weighted MRI of the cervical spine showing normal spinal cord caliber and signal intensity throughout the cervical segments. (D) Axial T2-weighted MRI of the cervical spine confirming preserved posterior column architecture and absence of the "inverted V" sign. All images were exported directly from the clinical software systems and compiled using Microsoft Word (Microsoft Corporation, Redmond, WA).

**Figure 4:**
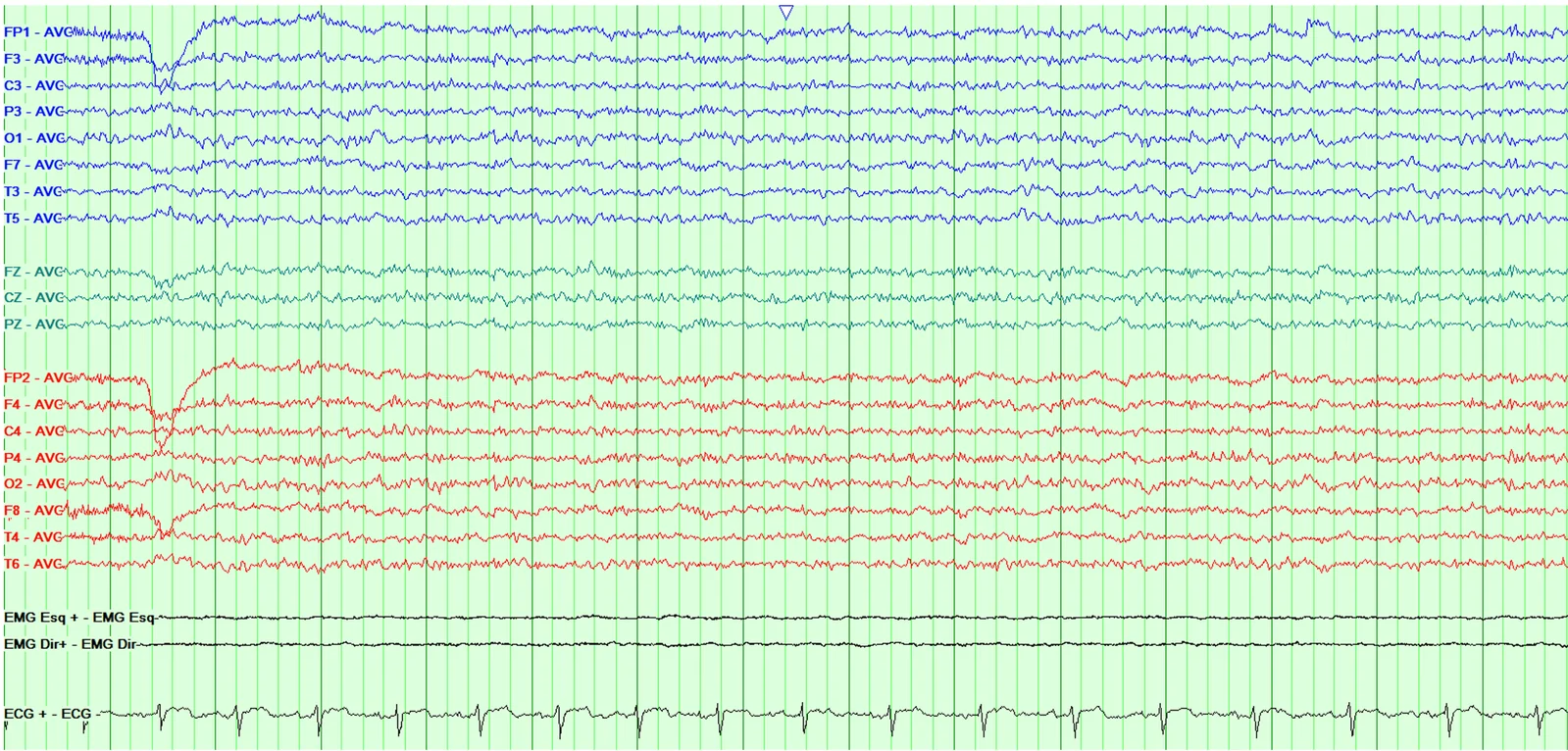
Representative segment of electroencephalogram (EEG) on day 5. A representative epoch selected from the complete standard 19-channel routine EEG recorded on day 5, demonstrating well-structured baseline electrogenesis. The trace exhibits moderate, medium-amplitude, posterior alpha activity with regular morphology (unstable frequency around 11 Hz) that is symmetric and reactive to eye opening. Sparse, symmetric anterior theta and beta activities are present, with no focal or generalized slowing, and an absence of epileptiform discharges. Image segment exported directly from the clinical EEG software and compiled using Microsoft Word (Microsoft Corporation, Redmond, WA).

Serial blood counts revealed a progressive decline in ANC from 3.25 × 10⁹/L on day 0 to a nadir of 1.06 × 10⁹/L on day 7, accompanied by relative lymphocytosis. Hemoglobin decreased to 11.4 g/dL without changes in MCV or hypersegmented neutrophils, and platelets fell to 222 × 10⁹/L. A baseline full blood count from two years prior was normal (ANC = 3.03 × 10⁹/L). Following three days of vitamin repletion, serum B12 increased to >2000 pg/mL and homocysteine decreased to 8.4 µmol/L on day 4. By day 12, all cell counts were within reference ranges. The dynamic trajectory of these laboratory parameters is illustrated in Figure 2.

Differential diagnosis

The abrupt symptom onset within one to three days of polysubstance exposure, fluctuating consciousness, disorientation, and profound amnesia favored a secondary substance-induced or toxic-metabolic etiology over a primary psychotic disorder. Severe sleep deprivation, known to trigger perceptual distortions, depersonalization, disorientation, and acute psychosis [7], was considered a major contributing factor rather than a sole explanation, as symptoms persisted for days after sleep was restored.

Autoimmune encephalitis, particularly anti-N-methyl-D-aspartate (NMDA) receptor encephalitis, was a critical differential consideration, given the acute psychiatric features, amnesia, fluctuating orientation, and reported orofacial dyskinesias in a young female [8]. Although formal exclusion via lumbar puncture and neuronal antibody panels was not performed (representing a notable limitation), the diagnosis was deemed unlikely due to normal MRI findings, normal EEG without slowing or epileptiform activity, absence of seizures, fever, or dysautonomia, and complete resolution by day 9 on abstinence, vitamin repletion, and low-dose antipsychotic therapy without immunotherapy.

A dissociative mechanism was considered regarding her selective amnesia (e.g., recognizing her aunt while failing to recognize her child). The subsequent account that she withheld recognition due to paranoia suggested a dissociative response nested within a broader toxic-metabolic state. Delirium secondary to infection, endocrine dysfunction, or structural lesions was excluded by negative inflammatory markers, normal metabolic panels, and unremarkable neuroimaging. A primary affective episode with psychotic features was considered less likely given the acute, substance-linked onset and the predominance of confusional and psychotic over affective symptoms.

Overall, the presentation was considered a multifactorial substance-induced psychotic disorder. MDMA-associated psychosis may persist for days to weeks [9,10], and MDMA may have contributed substantially. Two non-specific findings, however, raised suspicion of a relevant N2O contribution: laboratory evidence of functional cobalamin inactivation that reversed with repletion, the only objective marker of exposure; and prominent confusional and dissociative features, closer to reported N2O-related psychosis and to the effects of NMDA receptor antagonists than to stimulant psychosis [6,10]. Severe sleep deprivation can also produce similar features [7]. Applying the Naranjo adverse drug reaction probability scale to N2O, with MDMA and sleep deprivation scored as alternative causes, yielded a score of 4, indicating a possible association [11].

A detailed summary comparing the clinical, biochemical, and diagnostic features of the present case against previously published cases of nitrous oxide-associated psychosis is presented in Table 1.

**Table 1:** Comparison of clinical features between published cases of nitrous oxide-associated psychosis and the present case. MDMA: 3,4-methylenedioxymethamphetamine. Data for published cases were adapted from Garriga-Solé et al. [6]. The table was compiled by the authors.

Feature	Published cases (n = 20) [6]	Present case
Sex	17 male, 3 female	Female
Age	20-45 years	Early 20s
Exposure pattern	Regular daily use >3 months with recent escalation	Binge exposure over a single 4-day festival (6 group canisters)
Onset after last use	<24 to 72 hours in most reports	~24 hours to first symptoms; ED presentation on day 0
Co-ingested substances	Alcohol, cannabis, cocaine, MDMA	MDMA, episodic alcohol, severe sleep deprivation
Serum vitamin B12	Low or low-normal in most cases	270 pg/mL (low-normal)
Serum homocysteine	Elevated in most cases (up to 213 µmol/L)	25.2 µmol/L (normalized to 8.4 µmol/L)
Neurological signs	Common (myelopathy, ataxia, peripheral neuropathy)	Absent
Hematological findings	Macrocytic anemia, hypersegmented neutrophils	Normocytic anemia and transient neutropenia
Treatment	Abstinence, cobalamin supplementation, antipsychotics	Abstinence, cobalamin/folate, risperidone switched to aripiprazole
Length of hospital stay	A few days to 3 weeks (mean ~12 days)	12 days
Outcome	Full recovery in most; relapse in 3 cases	Resolution of psychotic symptoms with full insight at 3 weeks

Treatment and management

Initial management focused on safety using physical restraint and parenteral sedation (intramuscular haloperidol 5 mg and diazepam 10 mg, supplemented with as-needed promethazine and olanzapine). Involuntary status was converted to voluntary admission on day 9.

Empiric parenteral vitamin B12 (1 mg daily) and oral folic acid (5 mg daily) were initiated on day 1 upon suspecting N2O toxicity. Prophylactic enoxaparin (40 mg daily) was administered due to the established risk of venous thromboembolism associated with N2O-induced hyperhomocysteinemia [12,13]. On the recommendation of the national poison control center, high-flow oxygen (15 L/min via reservoir mask) was administered for three hours on day 2 and discontinued once arterial blood gases were confirmed normal.

Oral risperidone was titrated to 2 mg daily from day 3, alongside clonazepam 2 mg daily. As neutropenia was first detected on day 4 and worsened on day 6, risperidone was cross-tapered to oral aripiprazole 10 mg daily from day 7, which was well tolerated, and clonazepam was gradually tapered. Multidisciplinary care was coordinated across psychiatry, neurology, and internal medicine.

Outcome and follow-up

Neutrophil counts recovered within five days of transitioning to aripiprazole. Insight improved progressively from day 5, two days after risperidone initiation and one day after homocysteine normalization, and by day 9, psychotic symptoms had fully resolved. The patient gained full insight, attributed the event to substance exposure, and agreed to voluntary stay. A trial home leave on day 11 was successful, and she was discharged on day 12 on aripiprazole 10 mg daily and clonazepam 0.5 mg at bedtime, with community mental health follow-up.

At her three-week outpatient follow-up (day 21), she remained free of psychotic symptoms, reported only mild initial restlessness, had resumed physical exercise, maintained full insight, expressed no craving, committed to substance abstinence, and successfully returned to work. Physical examination revealed no neurological deficits or signs of myeloneuropathy. A comprehensive overview of the patient's clinical progression from presentation through recovery is detailed in Figure 1.

Patient's perspective

"I had an episode of psychosis where I lost touch with reality. I read hidden meaning into every gesture; if someone looked away, I thought it was a sign that they wanted to harm people close to me. It is difficult to explain, but everything felt strange and threatening. I now understand that these were psychotic symptoms brought on by the substances I used that weekend. I never want to experience that again, and I am completely committed to staying clean." (Statement provided directly by the patient and translated from Portuguese by the authors.)

## Discussion

This case presents distinct features regarding exposure duration and clinical demographics compared to existing literature. In a systematic review of 20 published N2O-associated psychosis cases, 17 patients were male, aged 20 to 45 years, with histories of heavy, chronic daily use extending over several months [6]. Furthermore, reported homocysteine levels in chronic users were generally higher (up to 213 µmol/L) [6]. In contrast, our patient’s exposure was compressed into a single festival weekend, with moderate hyperhomocysteinemia (25.2 µmol/L). This suggests that short-term binge exposure, combined with other substances and sleep deprivation, may be associated with severe neuropsychiatric symptoms, even when homocysteine is only moderately elevated.

The primary pathophysiological mechanism linking N2O to neurological damage involves oxidation of cobalamin's cobalt core, inactivating methionine synthase, elevating homocysteine, and impairing myelin synthesis [3-5,14]. However, this subacute metabolic pathway does not fully explain the rapid onset of acute dissociation and psychosis observed here. N2O also acts as a non-competitive NMDA receptor antagonist, sharing pharmacological properties with ketamine [15,16]. We hypothesize that acute NMDA antagonism, together with MDMA and sleep deprivation, may have contributed to the onset of symptoms, and functional cobalamin deficiency to their persistence. This remains speculative: the direct effects of N2O are short-lived, homocysteine normalized by day 4 while psychosis persisted until day 9, and antipsychotics were started concurrently.

Several practical considerations emerge from this case. First, total serum vitamin B12 is an unreliable marker of N2O toxicity because standard assays measure total rather than bioactive cobalamin [5,6]. Measurement of homocysteine (and methylmalonic acid where available) should be considered when N2O toxicity is suspected, ideally prior to vitamin administration. Second, obtaining accurate substance histories requires interviewing multiple collateral sources, as single informants may provide incomplete or contradictory information.

Regarding hematological findings, neutropenia was first detected on day 4, on the first repeat blood count, one day after starting low-dose risperidone, and worsened until day 7. N2O can cause bone marrow suppression [3,14], but this appears less likely here, as neutrophil counts were normal on admission, no hypersegmented neutrophils were seen, and the decline continued after homocysteine had normalized. Together with recovery after cross-titration to aripiprazole, this makes risperidone a plausible contributor [17,18]. However, causality cannot be established, given the unusually short latency, the concurrent fall in hemoglobin and platelets, and the use of other psychotropic drugs. Close monitoring of full blood counts allowed early detection and a timely switch to aripiprazole.

Limitations

Study limitations include the single-case design, concurrent MDMA use and sleep deprivation, lack of cerebrospinal fluid analysis and neuronal antibody testing, absence of methylmalonic acid measurement, reliance on collateral exposure estimates, and relatively short outpatient follow-up. Recovery coincided with vitamin repletion, antipsychotic treatment, abstinence, and restored sleep, so the main driver of recovery cannot be determined. Serum homocysteine was collected several days after the last exposure and after the first vitamin B12 dose, which may have underestimated its peak [19].

## Conclusions

Clinicians evaluating young adults presenting with acute psychosis or dissociative symptoms after attending festivals should specifically inquire about recreational nitrous oxide use, as it is undetectable on standard urine toxicology screens. This case suggests that short-term binge exposure, in combination with other substances and sleep deprivation, may be associated with severe neuropsychiatric presentations, even in the absence of chronic use or overt myeloneuropathy. Normal or low-normal serum vitamin B12 levels do not rule out functional cobalamin deficiency; measurement of serum homocysteine and, where available, methylmalonic acid should be considered. Finally, as both N2O and antipsychotics can affect blood counts, clinicians should remain alert to hematological changes during antipsychotic initiation in this setting, with investigation and monitoring when clinically indicated.
